# Preparation of spiroborate supramolecular and peapod polymers containing a photoluminescent ruthenium(ii) complex†

**DOI:** 10.1039/d3ra03940d

**Published:** 2023-08-23

**Authors:** Wako Matsumoto, Muneyuki Naito, Hiroshi Danjo

**Affiliations:** a Graduate School of Natural Science, Konan University 8-9-1 Okamoto, Higashinada Kobe 658-8501 Japan; b Department of Chemistry, Konan University 8-9-1 Okamoto, Higashinada Kobe 658-8501 Japan danjo@konan-u.ac.jp

## Abstract

The immobilization of functional metal complexes onto polymer supports remains one of the most important research areas. In this study, we prepared spiroborate supramolecular and peapod polymers containing a cationic photoluminescent ruthenium(ii) complex. The supramolecular polymer was obtained by mixing spiroborate cyclic trimer bearing homoallyl group and a ruthenium(ii) tris(bipyridyl) complex, and was further converted into the corresponding peapod polymer by olefin metathesis polymerization. The structure of these polymers was determined by ^1^H NMR, dynamic light scattering, inductively coupled plasma-atomic emission spectroscopy, energy dispersive X-ray analyses, and atomic force microscopy. The absorption and emission behaviors of the ruthenium(ii) complex were almost the same for the free form and the supramolecular polymer in the mixed solvent of *N*,*N*-dimethylformamide and chloroform, although the emission intensity decreased when the chloroform portion was increased. On the other hand, the hypsochromism was observed upon the emission of the ruthenium(ii) complex in the peapod polymer, probably due to the rigidochromic effect of the tight encapsulation by the peapod structure.

## Abstract 2

Spiroborate supramolecular and peapod polymers containing ruthenium(ii) tris(bipyridyl) complex were prepared, and their optical properties were evaluated.

## Graphical abstract

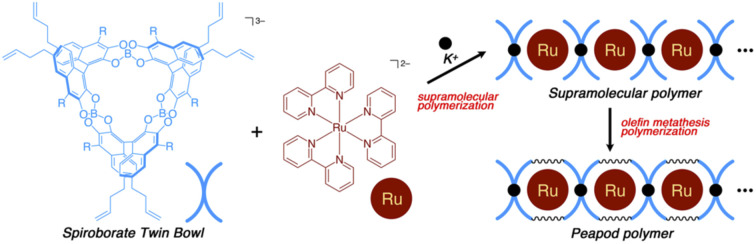

## Introduction

The development of polypyridyl ruthenium(ii) (Ru(ii)) complexes bound to polymer supports is one of the most important research topics because of their wide applications in such areas as photocatalysts or photoelectrodes for solar cells.^1^ The preparation of these polymer materials typically involves embedding the complex into the polymer main chain or side chain *via* a covalent bond.^2,3^ In both cases, introducing a functionality to the target complex is inevitable to fix the complex onto the polymer chain *via* a covalent bond.

We have reported the preparation of a supramolecular polymer *via* the iterative molecular recognition of twin-bowl-shaped spiroborate cyclic trimer with a tricationic iridium(iii) (Ir(iii)) complex.^4^ We have also demonstrated that the covalent bond formation between the adjacent spiroborate twin bowls in the supramolecular polymer led to the formation of a peapod-type nanotube structure in which the Ir(iii) complexes were encapsulated in the peapod cavity formed along with the polymer and topologically fixed onto the polymer main chain.^5^ In this system, no covalent bond formation is required for the fixation of the guest complex onto the polymer chain. The fact that the guest complex can be fixed without the need for any chemical transformation or introduction of functional groups used for bond formation would be quite advantageous for the development of functional organic materials. In this manuscript, we report the preparation of a spiroborate peapod polymer containing the tris(2,2′-bipyridine)ruthenium(ii) complex [Ru(bpy)_3_]^2+^. The Ru(ii) complex was topologically fixed onto the polymer chain in the peapod structure without any functionalization of the complex. The optical properties of the Ru(ii) complex inside the peapod polymer were also elucidated.

## Results and discussion

### Preparation of spiroborate supramolecular polymer M-SP and peapod polymer M-PP

**Scheme 1:**
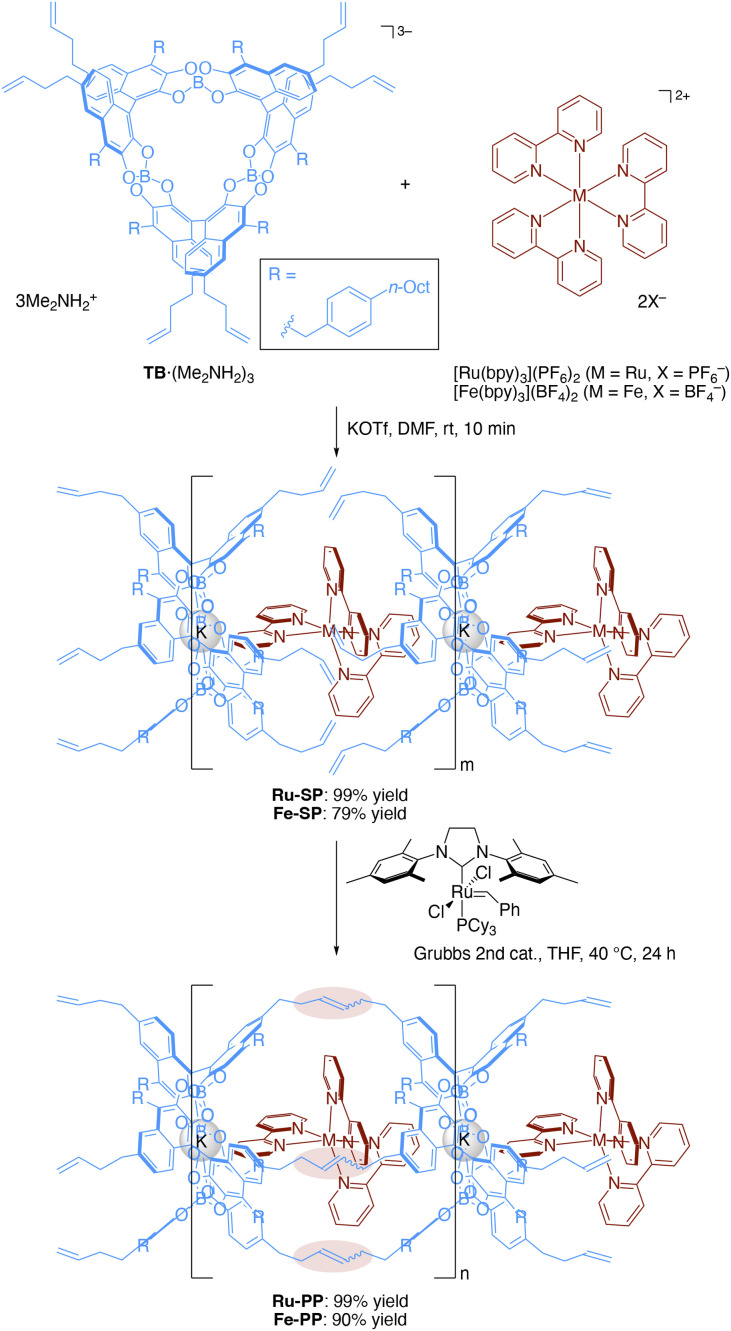
Preparation of supramolecular polymer M-SP and peapod polymer M-PP (M = Ru or Fe). The chemical yield was determined on the basis of the theoretical molecular weight, that was calculated for [TB·K·M(bpy)_3_] (for M-SP) or [TB·K·M(bpy)_3_–3C_2_H_4_] (for M-PP).

The preparation of the supramolecular polymer and the peapod polymer was carried out according to the previously reported procedures (Scheme 1).^4*b*,5^ The supramolecular polymer (Ru-SP) was obtained by mixing equimolar amounts of spiroborate twin bowl (TB·(Me_2_NH_2_)_3_), [Ru(bpy)_3_](PF_6_)_2_, and potassium trifluoromethanesulfonate (KOTf) in *N*,*N*-dimethylformamide (DMF), followed by precipitation with methanol. Potassium ion was employed to achieve a high degree of polymerization by adjusting the counter charges. Potassium ion was incorporated into the central cavity of TB^3−^ to form the stable complex [TB·K]^2−^, that could iteratively associate with [Ru(bpy)_3_]^2+^ without accumulating negative charges on the polymer chain. Ru-SP was then subjected to olefin metathesis polymerization using Grubbs 2nd generation catalyst, followed by precipitation with methanol and washing with acetonitrile to obtain the peapod polymer (Ru-PP). Fe-SP and Fe-PP, containing the iron(ii) (Fe(ii)) complex [Fe(bpy)_3_]^2+^ instead of [Ru(bpy)_3_]^2+^, were also prepared by the same procedure.

### Structural evaluation of Ru-SP and Ru-PP

**Fig. 1:**
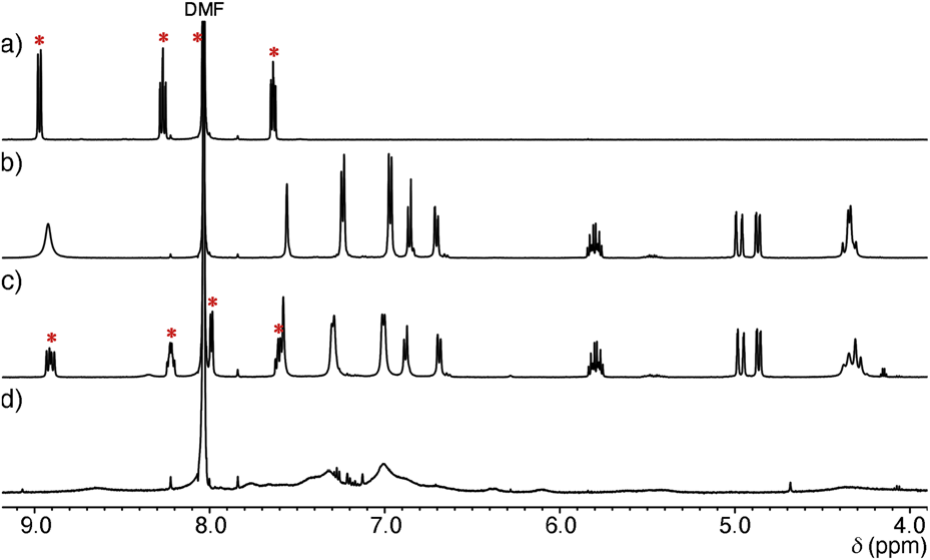
Partial ^1^H NMR spectra (500 MHz, 25 °C in DMF-*d*_7_) of (a) [Ru(bpy)_3_](PF_6_)_2_ (2 mM), (b) TB·(Me_2_NH_2_)_3_ (2 mM), (c) Ru-SP (3 mg mL^−1^), and (d) Ru-PP (3 mg mL^−1^). Signals marked by red asterisks are assigned to [Ru(bpy)_3_]^2+^.

Structural evaluation of Ru-SP and Ru-PP commenced with ^1^H NMR measurement. The proton signals of [Ru(bpy)_3_]^2+^ were shifted upfield and slightly broadened in the ^1^H NMR spectrum of Ru-SP, indicating the association of [Ru(bpy)_3_]^2+^ with [TB·K]^2−^ (Fig. 1c). In the ^1^H NMR spectrum of Ru-PP, all signals were markedly broadened, making it difficult to assign [Ru(bpy)_3_]^2+^ proton signals (Fig. 1d).^5^ In the peapod structure, the motion of the components would be restricted, resulting in the marked broadening of the proton signals.

**Fig. 2:**
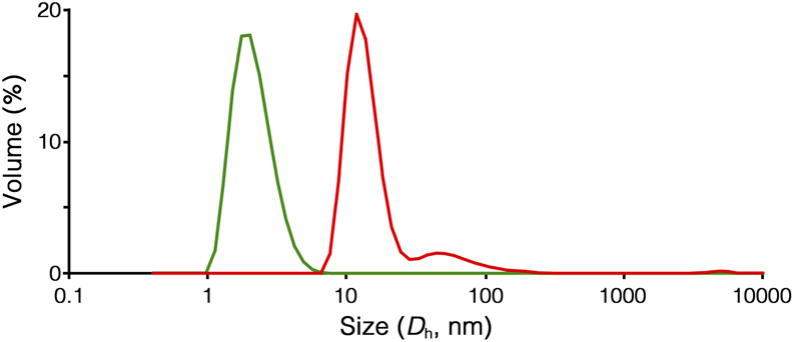
Size characterization of Ru-SP (green) and Ru-PP (red) using DLS (0.5 g L^−1^ in DMF, 20 °C).

The formation of the peapod structure in Ru-PP was further confirmed by the dynamic light scattering (DLS) measurement. The average hydrodynamic diameter (*D*_h_) of Ru-SP in DMF could only be detected at 2.2 nm owing to the dissociation of the polymer structure (Fig. 2). In the case of Ru-PP, the main peak was detected at *D*_h_ = 13.4 nm, together with a small amount of a larger component (*D*_h_ = 66 nm). This indicates that the covalent bond formation occurred between the adjacent TB^3−^s in the supramolecular polymer by the olefin metathesis polymerization.

The molar ratio of TB^3−^ to [Ru(bpy)_3_]^2+^ in the obtained supramolecular and peapod polymers was estimated by inductively coupled plasma-atomic emission spectroscopy (ICP-AES). The molar amounts were 0.97 and 0.24 mmol in 1 g of Ru-SP for boron (B) and Ru, respectively (Table 1, Fig. S1†), which meant that the molar ratio of TB^3−^ to [Ru(bpy)_3_]^2+^ was approximately 4 : 3 in Ru-SP, and around 25% of [Ru(bpy)_3_]^2+^ was lost during the purification process. On the other hand, ICP-AES analysis of Ru-PP gave the molar amounts of 0.83 and 0.27 mmol g^−1^ for B and Ru, respectively. In this case, the ratio of TB^3−^ to Ru was 1 : 1, which indicated an increase in the ratio of Ru compared with that in Ru-SP. To elucidate the cause of this increase in Ru, ICP-AES analysis was carried out for Fe-SP and Fe-PP. For Fe-SP, the molar amounts were 0.93 and 0.24 mmol g^−1^ for B and Fe, respectively, and the host/guest ratio was the same as that of Ru-SP. In the case of Fe-PP, the molar amounts were 0.82 and 0.21 mmol g^−1^ for B and Fe, respectively, and in addition, 0.077 mmol g^−1^ of Ru was detected. It was thought that Fe-PP contained a Ru center derived from the Grubbs 2nd generation catalyst used in the polymerization step. In this case, the atomic ratio of B, Fe, and Ru was estimated to be approximately 12 : 3 : 1, which meant that a single Ru center was bound to the peapod polymer per heptameric repeating unit, which was expected to contain four TB^3−^ units and three [Ru(bpy)_3_]^2+^ units. From these results, it was concluded that Ru-PP would also contain the catalyst-derived Ru center in a similar ratio. Energy dispersive X-ray (EDX) analysis of Ru-PP revealed that the atomic ratio of Ru to potassium (K) was approximately 55 : 35 (Fig. S2†). This indicates that around 35% of potassium ion was lost during the preparation of Ru-PP. In addition, phosphorus (P), probably derived from the Grubbs 2nd generation catalyst, was detected, although the molar ratio (Ru : P = 10 : 55) was smaller than expected.

**Table 1:** ICP-AES analysis of M-SP and M-PP (M = Ru or Fe)^a^

	Ru-SP	Ru-PP	Fe-SP	Fe-PP
B (mmol g^−1^)^b^	0.97	0.83	0.93	0.82
Ru (mmol g^−1^)	0.24	0.27	—	0.077
Fe (mmol g^−1^)	—	—	0.24	0.21
B/3 : M^c^	4 : 3	1 : 1	4 : 3	1 : 1

a Each sample was subjected to wet ashing using the mixed acid of HNO_3_ aq/HClO_4_ aq/H_2_SO_4_ and diluted with deionized water prior to analysis.

b Molar amount of atom per 1 g of polymer sample.

c Atomic ratio.

### Optical properties of Ru-SP and Ru-PP

**Fig. 3:**
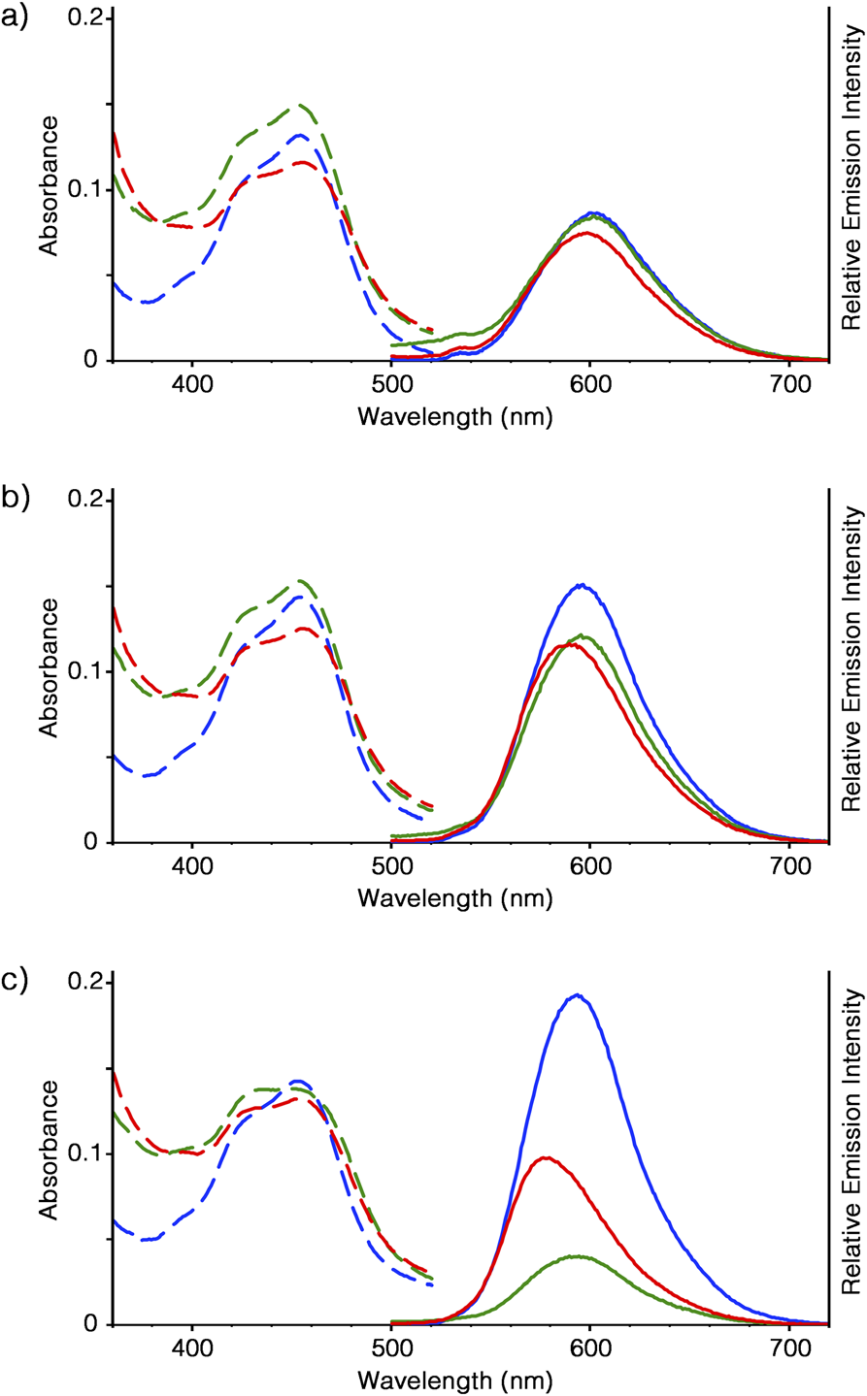
Absorption (left, dashed lines) and emission (right, solid lines, *λ*_ex_ = 460 nm) spectra of [Ru(bpy)_3_](PF_6_)_2_ (10 mM, blue), Ru-SP (40 mg L^−1^, green), and Ru-PP (40 mg L^−1^, red) in DMF/chloroform (v/v) = (a) 10 : 0, (b) 5 : 5, and (c) 1 : 9 at 25 °C.

The optical properties of [Ru(bpy)_3_]^2+^ in the forms of supramolecular polymer and peapod polymer were evaluated. From ICP-AES analysis, it was estimated that 40 mg of Ru-SP and Ru-PP incorporated approximately 9.6 and 8.4 μmol of [Ru(bpy)_3_]^2+^, respectively. In DMF solution, [Ru(bpy)_3_](PF_6_)_2_, Ru-SP, and Ru-PP showed similar absorption behavior (*ε*_max_ = 453 nm), and almost the same emission peaks were given by [Ru(bpy)_3_](PF_6_)_2_ and Ru-SP, as the metal to ligand charge transfer (MLCT) band at *λ*_max_ = 602 nm (Fig. 3a).^6^ As shown in the DLS analysis, Ru-SP was almost completely dissociated in DMF solution, and [Ru(bpy)_3_]^2+^ existed almost in the free form. Their emission maxima are the same even in DMF/chloroform mixed solvent (*λ*_max_ = 596 and 593 nm in DMF/chloroform = 5 : 5 and 1 : 9 (v/v), respectively, Fig. 3b and c). It was noteworthy that the emission intensity of Ru-SP decreased when the ratio of chloroform was increased. In the less polar solvent, the electrostatic interaction of TB^3−^ and [Ru(bpy)_3_]^2+^ became stronger, resulting in the quenching of the emission of [Ru(bpy)_3_]^2+^. On the other hand, a small blue shift was found for the emission spectrum of Ru-PP in comparison with that of [Ru(bpy)_3_](PF_6_)_2_. In DMF, the emission maximum of Ru-PP was found at *λ*_max_ = 598 nm, and the width of the emission shift from [Ru(bpy)_3_](PF_6_)_2_ (Δ*λ*_max_) was 4 nm. This hypsochromic effect became more pronounced when the ratio of chloroform in the solvent was increased, and Δ*λ*_max_ finally reached up to 16 nm in DMF/chloroform = 1 : 9 (Fig. 3c). We anticipated that this blue shift was caused by the rigidochromic behavior of [Ru(bpy)_3_]^2+^ in Ru-PP.^7–9^ The guest complex in the peapod polymer was tightly encapsulated in the cavity formed by the two adjacent spiroborate twin bowls and lost its motional freedom. In this case, the excited state of [Ru(bpy)_3_]^2+^ would have an asymmetric charge distribution, and reorientation of the distribution took place very slowly inside the cavity. This led to the long lifetime of the Franck–Condon excited state, and the successive emission occurred directly from this unrelaxed state.

### AFM observation of Ru-SP and Ru-PP

**Fig. 4:**
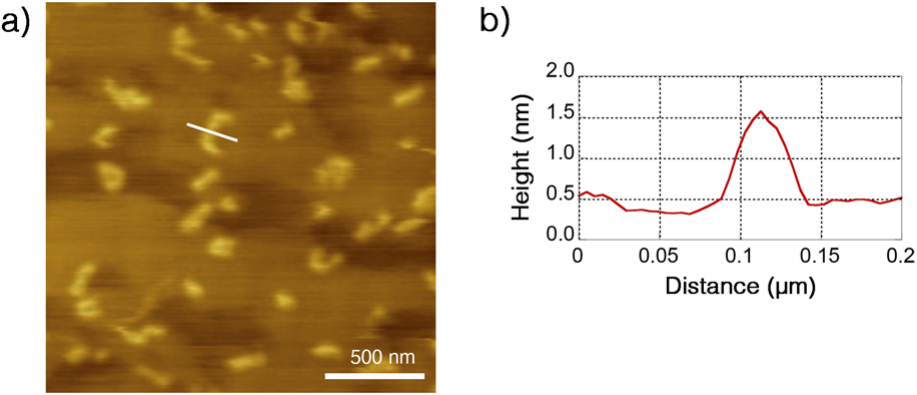
(a) Dynamic-mode AFM image (2.0 × 2.0 μm^2^ on a HOPG substrate) of Ru-PP and (b) height profile on a cross-section of the white line.

The structure of Ru-SP and Ru-PP was evaluated by atomic force microscopy (AFM). On a highly oriented pyrolytic graphite (HOPG) substrate, no specific aggregation morphology could be found for Ru-SP (Fig. S3†). On the other hand, Ru-PP gave some island-shaped aggregates, and the thickness were estimated to be about 1.5 nm from the cross-sectional height profile (Fig. 4). Since this value shows good agreement with the thickness of the spiroborate peapod polymer reported previously, those aggregates could be regarded as the monolayers of the peapod polymer.^5^

## Conclusions

Spiroborate supramolecular and peapod polymers were prepared by using photoluminescent Ru(ii) complex [Ru(bpy)_3_]^2+^ as the cationic monomer component. The spiroborate supramolecular polymer was easily obtained by mixing homoallylated spiroborate twin bowl and [Ru(bpy)_3_](PF_6_)_2_ in DMF, followed by precipitation with methanol. This supramolecular polymer was then converted into the spiroborate peapod polymer *via* olefin metathesis polymerization by using Grubbs 2nd generation catalyst. The formation of these polymers was confirmed by ^1^H NMR, ICP-AES, and DLS analyses. ICP-AES analysis revealed that these polymers were composed of spiroborate twin bowl and [Ru(bpy)_3_]^2+^ in the ratio of approximately 4 : 3, and residual Grubbs catalyst-derived Ru component was also detected in the peapod polymer. The optical properties of [Ru(bpy)_3_]^2+^ in the forms of supramolecular and peapod polymers, and as free form [Ru(bpy)_3_](PF_6_)_2_ were evaluated. In the supramolecular polymer, [Ru(bpy)_3_]^2+^ showed almost the same absorption and emission behavior as the free form in DMF, although the emission intensity was decreased when the ratio of chloroform in the solvent was increased because the enhanced interaction between Ru complex and the twin bowl resulted in emission quenching. On the other hand, a significant blue shift of the emission of [Ru(bpy)_3_]^2+^ in the form of the peapod polymer was observed, probably because of the rigidochromic effect caused by the tight encapsulation inside the peapod cavity. In AFM observation, some island-shaped monolayer aggregates were observed for the peapod polymer.

## Experimental section

### General

^1^H NMR spectra were recorded on an Agilent Unity INOVA 500 at 25 °C. Chemical shifts were reported in *δ* ppm referenced to an internal tetramethylsilane standard.

### Materials

Spiroborate twin bowl (TB·(Me_2_NH_2_)_3_) was prepared according to the reported procedure.^5^ All commercially available reagents were used without further purification. The chemical yield was determined based on the theoretical molecular weight, that was calculated for [TB·K·M(bpy)_3_] for M-SP or [TB·K·M(bpy)_3_–3C_2_H_4_] for M-PP.

### Synthetic procedures and characterization

#### Preparation of Ru-SP: a typical procedure

In a 20 mL flask, TB·(Me_2_NH_2_)_3_ (424.4 mg, 160.0 μmol), [Ru(bpy)_3_](PF_6_)_2_ (137.7 mg, 160.0 μmol), and potassium trifluoromethanesulfonate (36.2 mg, 192.0 μmol) were dissolved in 4.0 mL of DMF at room temperature, and the mixture was stirred at intact temperature for 10 min. Methanol (50 mL) was then added to the solution, and the resulting brown precipitate was collected by centrifugation. The precipitate was washed with methanol and dried under reduced pressure to give Ru-SP as a brown solid (480.6 mg, 99% yield); ^1^H NMR (500 MHz, DMF-*d*_7_) *δ* (ppm) 8.92 (d, *J* = 8 Hz, 3H), 8.90 (d, *J* = 8 Hz, 3H), 8.23 (d, *J* = 8 Hz, 3H), 8.21 (d, *J* = 8 Hz, 3H), 7.99 (d, *J* = 6 Hz, 6H), 7.61 (t, *J* = 8 Hz, 6H), 7.58 (s, 6H), 7.29 (d, *J* = 6 Hz, 12H), 7.00 (d, *J* = 6 Hz, 12H), 6.88 (d, *J* = 9 Hz, 6H), 6.69 (d, *J* = 9 Hz, 6H), 5.79 (ddt, *J* = 17, 11, 7 Hz, 6H), 4.97 (dd, *J* = 17, 2 Hz, 6H), 4.86 (dt, *J* = 11, 1 Hz, 6H), 4.40–4.25 (m, 12H), 2.62 (d, *J* = 8 Hz, 12H), 2.49 (d, *J* = 8 Hz, 12H), 2.26 (q, *J* = 8 Hz, 12H), 1.52 (br s, 12H), 1.32–1.18 (m, 60H), 0.86 (t, *J* = 7 Hz, 18H).

#### Fe-SP

79% yield as a purple solid; ^1^H NMR (500 MHz, DMF-*d*_7_) *δ* (ppm) 8.92 (d, *J* = 6 Hz, 3H), 8.88 (d, *J* = 6 Hz, 3H), 8.30–8.22 (m, 6H), 7.68–7.52 (m, 18H), 7.34–7.22 (m, 12H), 7.01 (d, *J* = 7 Hz, 12H), 6.88 (d, *J* = 9 Hz, 6H), 6.69 (d, *J* = 9 Hz, 6H), 5.88–5.72 (m, 6H), 4.96 (d, *J* = 17 Hz, 6H), 4.86 (d, *J* = 10 Hz, 6H), 4.50–4.10 (m, 12H), 2.65–2.55 (m, 12H), 2.54–2.42 (m, 12H), 2.30–2.20 (m, 12H), 1.57–1.46 (m, 12H), 1.35–1.15 (m, 60H), 0.86 (t, *J* = 7 Hz, 18H).

#### Preparation of Ru-PP: a typical procedure

In a 20 mL 2-necked round-bottomed flask, Ru-SP (237.4 mg, 76.0 μmol) and Grubbs 2nd catalyst (32.2 mg, 38.0 μmol) was dissolved in THF, and stirred at 40 °C for 24 h. After allowed to room temperature, methanol (50 mL) was added to the solution, and the brown precipitate was collected by centrifugation. The precipitate was washed with acetonitrile and dried under reduced pressure to give Ru-PP as a dark brown solid (231.7 mg, 99% yield); ^1^H NMR (500 MHz, DMF-*d*_7_) *δ* (ppm) 8.64 (br s), 7.77 (br s), 7.32 (br s), 7.01 (br s), 6.38 (br s), 6.10 (br s), 5.48 (br s), 4.36 (br s), 2.49 (br s), 1.51 (br s), 1.25 (br s), 0.86 (br s).

#### Fe-PP

90% yield as a dark purple solid; ^1^H NMR (500 MHz, DMF-*d*_7_) *δ* (ppm) 7.59 (br s), 7.32 (br s), 6.99 (br s), 6.36 (br s), 5.78 (br s), 5.38 (br s), 4.95 (br s), 2.50 (br s), 1.52 (br s), 1.25 (br s), 0.86 (br s).

## Author contributions

WM performed syntheses. WM and HD performed solution studies. MN performed EDX and AFM studies. All authors contributed to experiment design and interpretation. HD and MN wrote the manuscript. All authors proofread and improved the manuscript.

## Conflicts of interest

There are no conflicts to declare.

## Supplementary Material

RA-013-D3RA03940D-s001
