# Supplementary material for: Preparation of spiroborate supramolecular and peapod polymers containing a photoluminescent ruthenium(ii) complex
Source: RSC Adv. 2023 Aug 23;13(36):25002–6. doi: 10.1039/d3ra03940d (PMC10445220; doi:10.1039/d3ra03940d)
Supplement: RA-013-D3RA03940D-s001 [file RA-013-D3RA03940D-s001.pdf]

Electronic Supplementary Material (ESI) for RSC Advances.
This journal is © The Royal Society of Chemistry 2023

Preparation of spiroborate supramolecular and peapod polymers containing a photoluminescent ruthenium(II) complex

Supporting Information

Wako Matsumoto,[a] Muneyuki Naito,[b] and Hiroshi Danjo*[b]

[a]Graduate School of Natural Science and [b]Department of Chemistry, Konan University, 8-9-1 Okamoto, Higashinada, Kobe 658-8501, Japan

Table of Contents

• Spectral copies of ^{1}H NMR S1
• Experimental details of Fig. 1–4, S1–S3 S3

Ru-SP, ^{1}H NMR (500 MHz, 3 mg/mL in DMF-d_7, 25 °C)

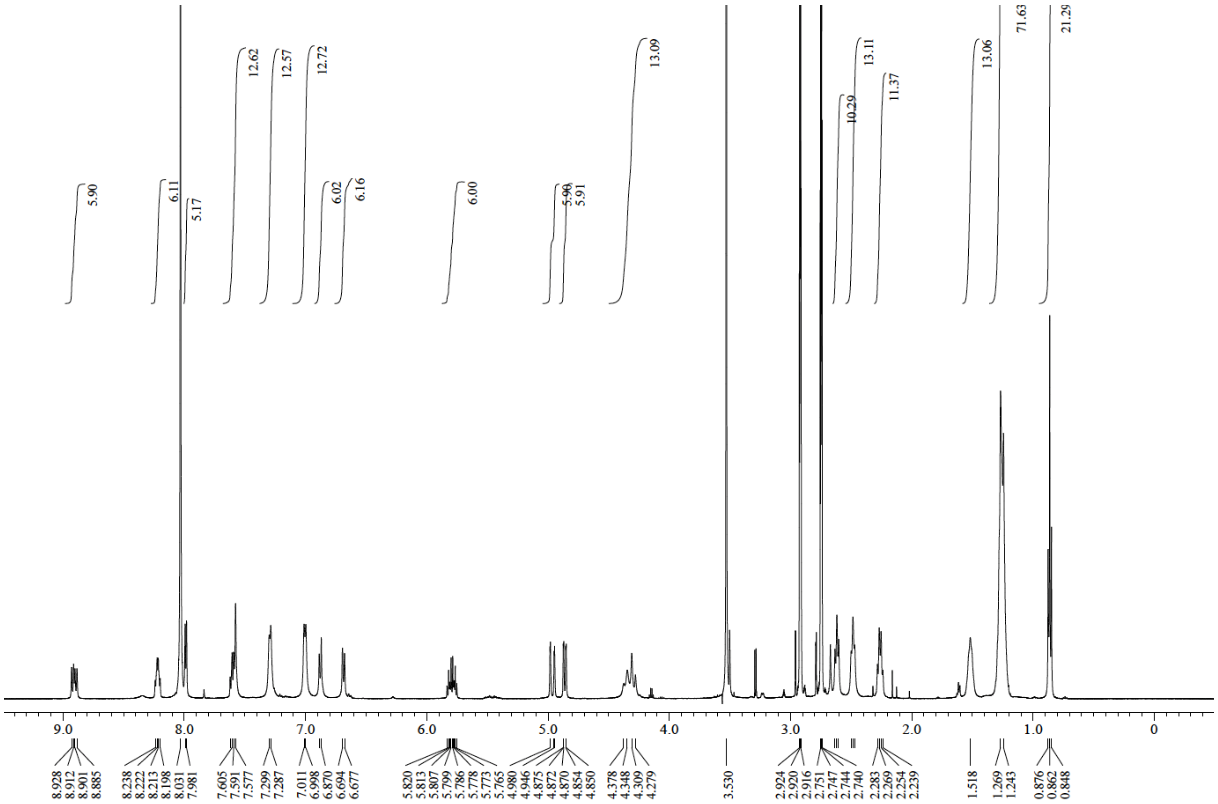

Ru-PP, ^{1}H NMR (500 MHz, 3 mg/mL in DMF-d_7, 25 °C)

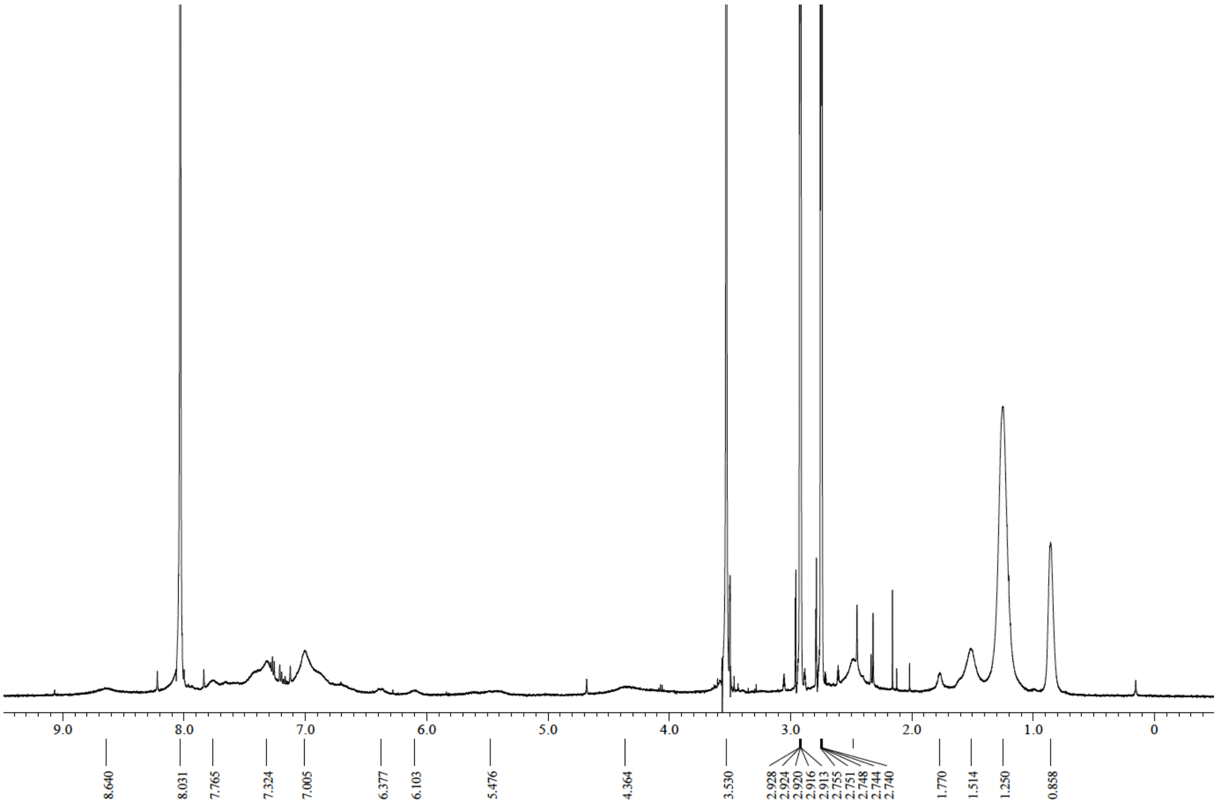

Fe-SP, ^{1}H NMR (500 MHz, 3 mg/mL in DMF-d_7, 25 °C)

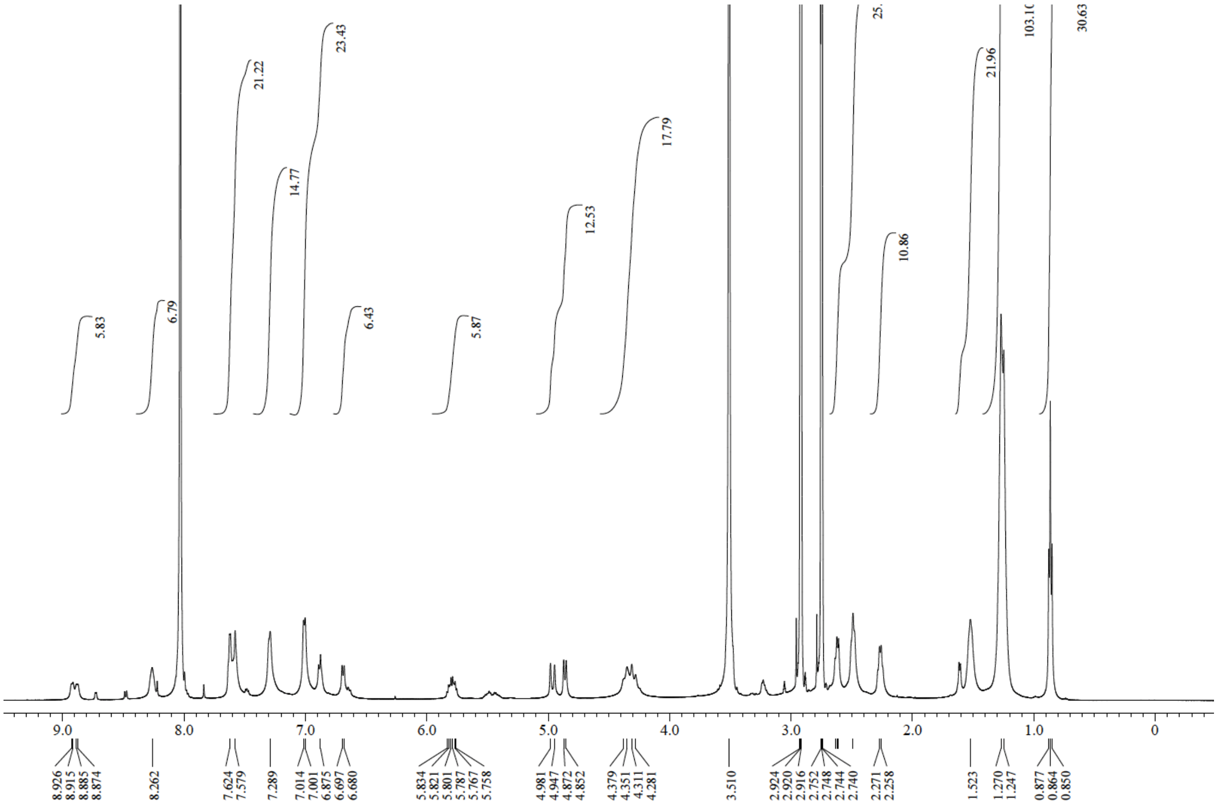

Fe-PP, ^{1}H NMR (500 MHz, 3 mg/mL in DMF-d_7, 25 °C)

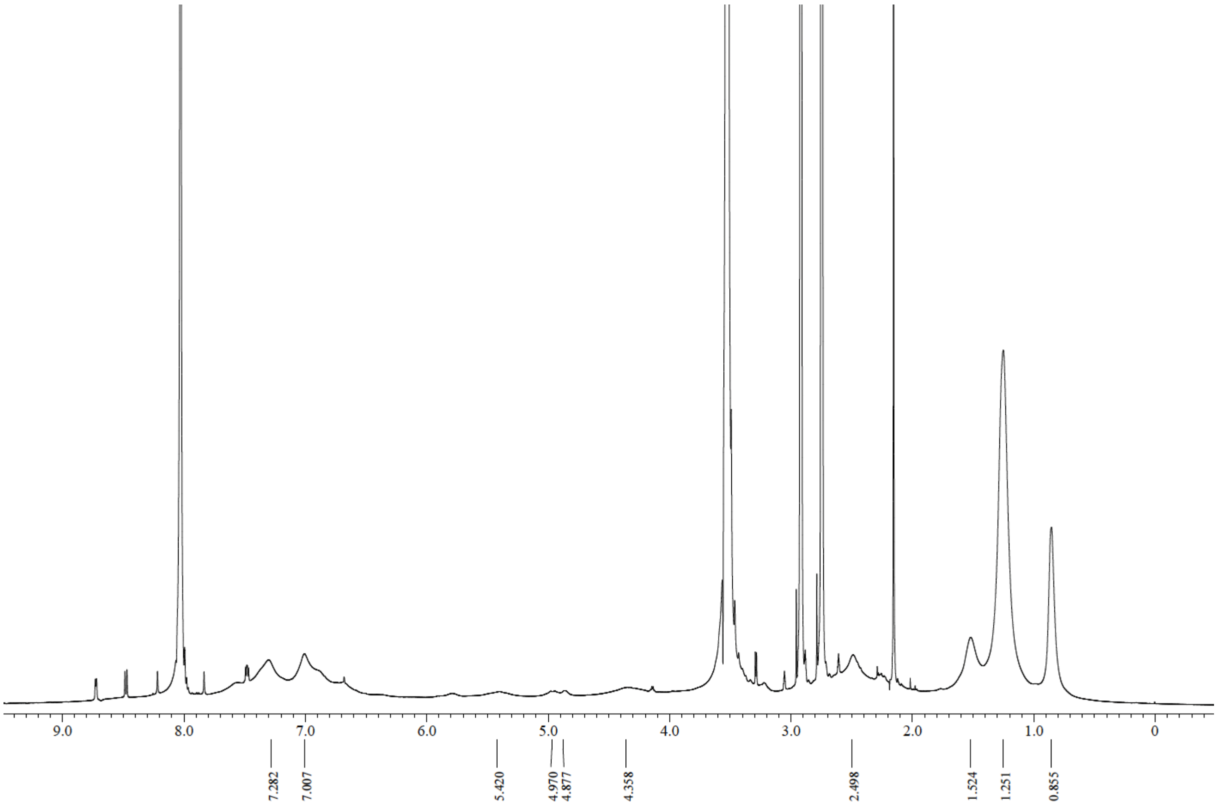

^{1}H NMR experiment (Fig. 1)

^{1}H NMR experiments were performed on an Agilent UNITY INOVA 500 (500 MHz) at 298 K. In preparation of sample solutions, $[Ru(bpy)_3](PF_6)_3$ (2.0 μmol) (for (a)), **TB**·$(Me_2NH_2)_3$ (2.0 μmol) (for (b)), **Ru-SP** (3.0 mg) (for (c)), or **Ru-PP** (3.0 mg) (for (d)) was dissolved in 1.0 mL of DMF-d_7 in a vial, and transferred into a 5 mm NMR tube with adjusting 50 mm of solution height.

Dynamic light scattering (DLS) experiment (Fig. 2)

Dynamic light scattering experiments were performed using a Malvern Instruments Zetasizer-Nano ZS equipped with a 4 mW He-Ne laser (633 nm wavelength) at a fixed detector angle of 90°. Measurements were performed at 20 °C. For data analysis, the viscosity and refractive index of DMF at 20 °C (0.9200 mPa·s and 1.431, respectively) were used. The measurements were performed in square quartz cell. The autocorrelation functions of the backscattered light fluctuations were analyzed (Stokes-Einstein) using the DTS v4.20 software (Malvern) that provided the hydrodynamic diameter (D_h), polydispersity and the size distribution. In preparation of sample solutions, 1.0 mg of **Ru-SP** or **Ru-PP** was dissolved in 2.0 mL of DMF in a vial, and transferred into a 10 mm glass cuvette.

UV-vis and photoluminescence analyses (Fig. 3)

UV-vis spectra were recorded on a JASCO V-660. Each sample concentration was adjusted to 10 μM (for $[Ru(bpy)_3](PF_6)_2$) or 40 mg/L (for **Ru-SP** and **Ru-PP**) in each solvent. The measurements were performed in square quartz cell at 25 °C. Photoluminescence spectra were recorded on a SHIMADZU RF-5300PC. Each sample concentration was adjusted to 10 μM (for $[Ru(bpy)_3](PF_6)_2$) or 40 mg/L (for **Ru-SP** and **Ru-PP**) in each solvent. The measurements were performed in square quartz cell at 25 °C.

Atomic force microscopic (AFM) observation (Figs. 4 and S3)

Atomic force microscopy (AFM) studies were performed with a Shimadzu SPM-9700 microscope. All experiments were carried out in dynamic mode at ambient atmosphere. A silicon cantilever was used with a resonance frequency of 150 kHz. The sample was prepared by dropping DMF/chloroform (ca. 1:20 (v/v)) solution of **Ru-SP** or **Ru-PP** (1.4 mg/L) on a HOPG substrate.

ICP-AES analysis (Fig. S1)

ICP-AES analyses were carried out on a SPECTRO ARCOS FHX22 inductively coupled plasma-optical emission spectrometer equipped with a radial torch, linear CMOS array detector, and nebulizer chamber for sample introduction. The plasma conditions used were an rf power of 1.20 kW applied to the plasma and flow rates of 13 L/min for the plasma gas, 0.8 L/min for the auxiliary gas, and 0.8 L/min for the nebulizer gas. The pump ratio of the sample uptake was 1.8 mL/min for each of three replicate scans. Elemental standard solutions to ICP-AES optimization were purchased from FUJIFILM Wako pure chemicals (for B and Fe) or Acros Organics (for Ru). In preparation of sample solution, 1.0 mg of each sample was subjected to wet ashing using the mixed acid of 60% HNO_3 aq, 60% $HClO_4$ aq, and conc. H_2SO_4 (10:5:3 volume ratio) and diluted with deionized water with adjusting total volume to 10 mL necessary to perform the ICP-AES determinations. Plasma emission was detected at 249.773 nm (for B), 240.272 nm (for Ru), or 261.187 nm (for Fe).

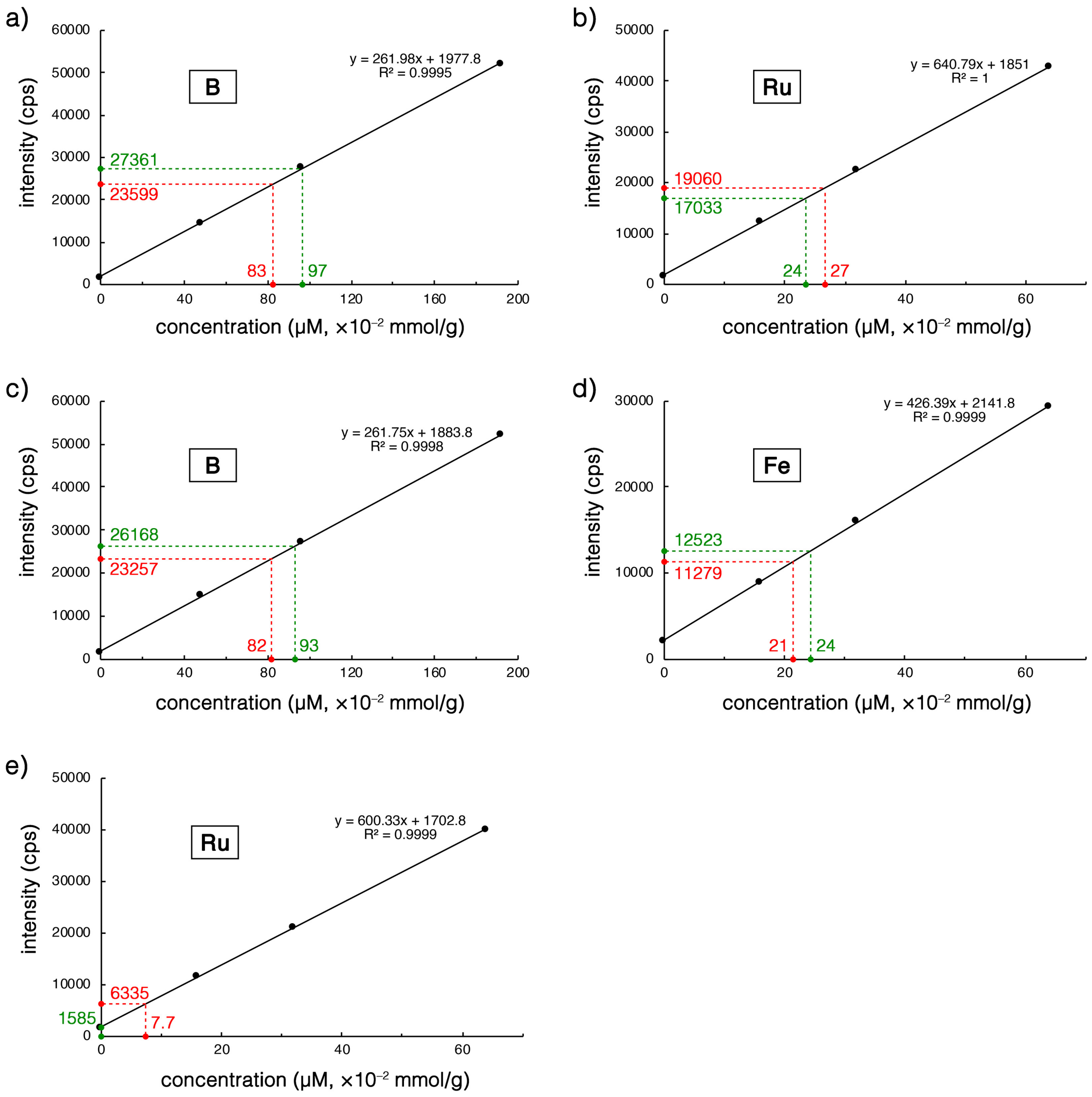

Fig. S1 ICP-AES analysis of **Ru-SP**, **Ru-PP**, **Fe-SP**, and **Fe-PP**. Boron (a) and ruthenium (b) of **Ru-SP** (green) and **Ru-PP** (red), and boron (c), iron (d), and ruthenium (e) of **Fe-SP** (green) and **Fe-PP** (red).

Energy dispersive X-ray analysis (Fig. S2)

Energy dispersive X-ray (EDX) measurements were performed using Oxford Instruments INCA X-Max 80 detector installed in a JEOL JEM-2100 transmission electron microscope. PP powders dispersed in methanol were crushed in an agate mortar, and the obtained PP fragments were deposited on a Cu microgrid. EDX spectra were collected with 200 kV electron beam with a size of approximately 300 nm. For quantitative data analysis, Cliff-Lorimer method was employed.

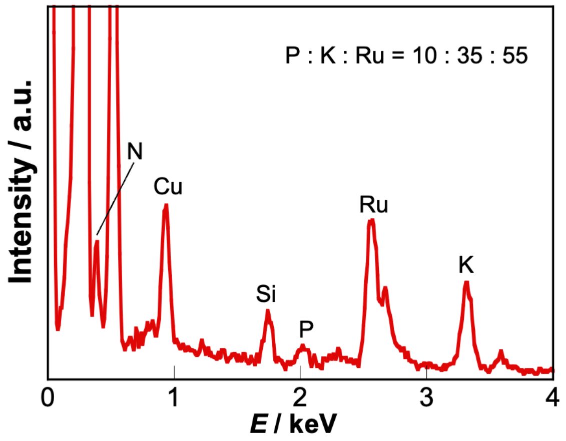

Fig. S2 EDX spectrum of **Ru-PP**.

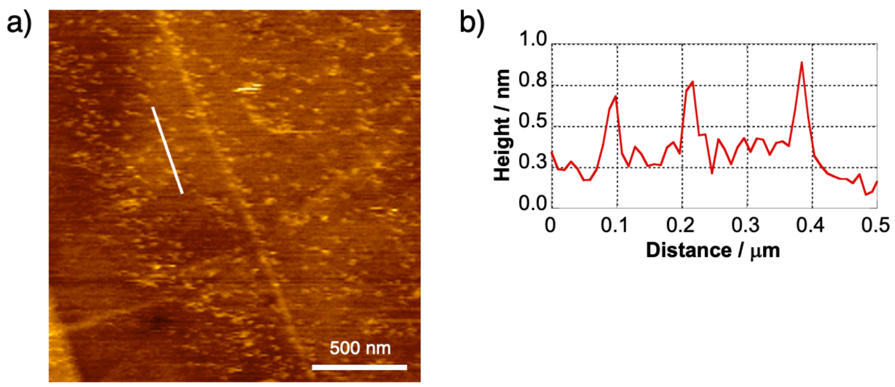

Fig. S3 Dynamic-mode AFM image (2.0 × 2.0 µm^2 on a HOPG substrate) of **Ru-SP** (a) and height profile on a cross-section of the white line (b).
